# Selecting Cost-Effectiveness Methods for Health Benefits Package Design: A Systematic Approach

**DOI:** 10.34172/ijhpm.8562

**Published:** 2025-03-30

**Authors:** Cassandra Nemzoff, Sedona Sweeney, Rob Baltussen, Anna Vassall

**Affiliations:** ^1^London School of Hygiene and Tropical Medicine, London, UK.; ^2^Center for Global Development, Washington, DC, USA.; ^3^Radboud University Medical Center, Nijmegen, The Netherlands.

**Keywords:** Health Benefits Package, Health Technology Assessment, Economic Evaluation, Cost-Effectiveness

## Abstract

**Background:**

Cost-effectiveness (CE) is a common prioritization criterion in health benefits package (HBP) design. However, to assess CE is a time- and data-demanding process, so most HBP exercises rely wholly or partially on global evidence. Extensive investment has been made in analyses, models, and tools to support cost-effectiveness analyses (CEAs) for HBPs. However, little attention has been paid to how national HBP assessors should both understand and select CE estimates. A structured, national process to select assessment methods is essential for ensuring the accuracy, ownership, and transparency of HBP design. This can be supported by "adaptive" health technology assessment (aHTA) principles, which focus on structured methodological choices based on the time, data, and capacity available. The objective of this paper was to apply aHTA framing to CEA methods selection for HBPs, and to make recommendations on how countries may consider systematically making these choices going forward.

**Methods:**

We first reviewed the definitions and categorization of different aHTA methods. We then conducted a scoping review of previous HBP assessments to understand how CEA methods used in HBPs fit into the aHTA framework, and a follow-up survey of authors to fill gaps. Results of the literature review and survey were interpreted and narratively synthesized.

**Results:**

We found that previous HBP assessments used four aHTA methods, sometimes simultaneously: expert opinion (n=3/20), review (n=12/20), model adaptation (n=6/20), and new model (n=2/20). The literature review and survey found that aHTA methods for HBPs take between 1-13 months; require different data sources depending on the method(s) used; and generally, require capacity in health economics, medicine, public health, and CE modelling. We supplement our report with a discussion of key considerations for methods selection.

**Conclusion:**

Trading off time, data, and capacity needs for different CE assessment methods can help to support structured, local design of HBP assessments.

## Background

**Key Messages**

**Implications for policy makers**

- Many tools and data sources exist to support health benefits package (HBP) prioritization, but little guidance is available on how to select between them.
- This study is the first of its kind to classify methods for assessing cost-effectiveness (CE) in HBP design and the time, data, and capacity required for each.
- Our framework provides practical guidance and information to enable national policy-makers to select the optimal methods for their context.

**Implications for the public**

Since the 1993 World Development Report, many tools and resources have been developed to support the prioritization of health benefits packages (HBPs). HBPs detail which health services are provided, to which members of the public, and at what cost. A cornerstone of HBPs is cost-effectiveness analysis (CEA), which helps to allocate resources based on the balance of their costs and the benefits they provide. While HBP prioritization has been ongoing for years, no resource to date has summarized the potential methods for assessing cost-effectiveness (CE) and the trade-offs between them. This paper presents a framework, which can be used by national stakeholders, to determine which methods are best suited for their context.

Many countries design or refine their health benefits packages (HBPs) for either government-funded or social insurance as part of the road to universal health coverage (UHC). This often draws on principles of health technology assessment (HTA). HTA is a process to facilitate evidence-based priority setting which has historically focused on cost-effectiveness analysis (CEA) to prioritize health interventions.^1,2^ In the context of HBPs, cost-effectiveness (CE) is used to improve allocative efficiency.^3,4^ However, a *de novo* CEA on a single intervention can take up to a year to complete.^5^ Prioritizing HBPs can be done using this type of “incremental” analysis, assessing one intervention at a time, or “sectoral” analysis, which assesses many interventions simultaneously.^3^ For the latter which sometimes requires sourcing cost-effectiveness ratios (CERs) for more than 100 interventions at a time, this is not feasible. Instead, principles of “adaptive” health technology assessment (aHTA) can be applied by employing global evidence on CE to adapt for time, data, and capacity constraints.^6^

 Various databases, tools, and resources of evidence, also known as “global public goods” (GPGs) have been developed to make accessible and, in some cases, synthesize this global evidence. These include the Tufts CEA registry, a database that synthesizes more than 12 000 global CEAs; World Health Organization (WHO) CHOosing Interventions that are Cost-Effective (WHO-CHOICE), a set of models to evaluate CE for 20 disease areas; Disease Control Priorities (DCP), nine volumes reporting systematic reviews on the evidence for cost-effective interventions for low- and middle-income countries (LMICs); and more recently, meta-regressions, which predict incremental cost-effectiveness ratios (ICERs) for many countries at once using regression analysis based on existing CEAs.^7-12^ However, there is little guidance on how to select which of these resources to use, which could be facilitated by clearly considering the trade-offs in time, data, and capacity requirements of different methods.

 Centering the selection and use of CEA estimates within national assessment teams is important for supporting the institutionalization of legitimate, transparent priority setting processes.^13^ Like many global health projects,^14^ the current GPGs are predominately produced by development assistance agencies or non-governmental organizations. Currently efforts are being made to regionalize priority setting methods such as the continental framework for Africa,^15^ but it is possible that HBP methods selection is influenced by technical partners who are producers of specific GPGs. While these tools and technical assistance may support capacity strengthening, it is important that the selection process for each CEA estimate remains centered and driven by the local decision context and actors, and not predetermined by whichever funder or group is a technical partner.

 The inspiration for this work was co-authors’ experience working with policy-makers and stakeholders involved in HBP design for the first time, who we observed are strong in articulating which interventions they want to prioritize, but many need help articulating how they might be able to prioritize them by outlining methodological options clearly and articulating trade-offs between them. The objective of this paper was thus to apply aHTA framing to CEA methods selection for HBPs, and to make recommendations on how countries may consider making these choices going forward in a systematic way.

## Methods

 Our methods were carried out in three steps: (1) defining aHTA and its characteristics; (2) conducting a scoping review of existing CEA methods for HBPs to identify aHTA methods used in HBP design; and (3) aligning the scoping review and survey to the aHTA framework.

###  Step 1: Defining Adaptive Health Technology Assessment and its Characteristics 

 Our starting point was the concept of aHTA, which is an approach to HTA that adapts for time, data, and capacity constraints and makes use of evidence from other jurisdictions where possible.^6^ Time refers to the analytical time for assessment; data includes all primary or secondary inputs required for assessment; and capacity reflects the technical and applied skills needed from an assessment team to conduct an analysis. A recent systematic review characterized the aHTA methods undertaken by global HTA agencies into a framework with five methods. These included de-facto HTA, which collates key characteristics of an intervention including registration, pricing, and international HTA decisions; rapid reviews, which review and synthesize existing literature; rapid manufacturer submissions, which review evidence produced in manufacturers’ dossiers; transfers, which use existing transferability frameworks to transfer economic evaluations from one jurisdiction to another; and rapid CEA, which conducts de novo CEA using pragmatically sourced data.^6^ Overall, aHTA can reduce the analytical time needed for analysis and has the potential to improve the efficiency of the priority setting process.^6,16^

 These aHTA methods were defined based on HTA agencies’ methods, which typically conduct incremental analyses. However, HBP assessments more often use “sectoral analyses” to prioritize many interventions at once. Assessments for HBPs must also adjust for time, data, and capacity constraints, are increasingly adopting HTA-like procedures.^16^ Indeed, the aHTA systematic review mentions that there is a need to better categorize aHTA methods for HBP design.^6^ We therefore sought to better understand how aHTA principles and methods have been applied in HBP design.

###  Step 2: Scoping Review and Survey

####  Scoping Review

 A rapid scoping review was designed to identify CE assessment methods used for HBPs globally. The search combined the concepts “health benefits package,” “cost-effectiveness,” and “assessment” (Supplementary file 1), with the aim of mapping them to the aHTA framing outlined in Step 1. Our search was run in MEDLINE (Ovid) in July 2022, and re-run in December 2023. Papers identified through snowballing and those known to co-authors were also reviewed for inclusion. This approach achieved saturation, in that the search is replicable, and further searching would obtain no additional unique methods for assessing CE in HBPs.^17^

 Papers that reported the assessment of CE in the context of HBP design or prioritization from individual countries or regions were included. To eliminate outdated methods, we included any papers from 2010 to present. CEAs for a single intervention; systematic reviews or broad overviews of CE methods; and commentaries or papers focused exclusively on lessons learned were excluded. Extraction for each paper was completed in Microsoft Excel by the first author and was split into three parts: methods; time and capacity; and data.

 For methods, we extracted the name of the method, a summary of how CE was assessed, and how many interventions were assessed. For methods that required transferring CEA estimates from other countries, criteria used to determine transferability were documented. We used the Welte knock-out criteria as a guide, which include geographic relevance, relevance of the intervention and comparator, and quality.^18^ For methods that required re-parameterizing CERs (eg, with unit cost, resource use, effect size, burden of disease), any adjustments made to CERs to account for bias from this approach were documented.

 Time to complete the assessment was documented for each method as described in the paper. Capacities needed were captured using the international Decision Support Initiative HTA capacity assessment questionnaire as a guide.^19^ This included listing any university-level skills reported (health economics/econometrics, economics, clinical/medical, pharmacy, epidemiology, public health) or applied skills (CEA, budget impact analysis, clinical evidence synthesis, policy analysis, HBPs, ethics and values in policy decision-making, evidence to policy translation) reported. Finally, data included a list of global or national sources of CE studies or parameters used.

####  Survey of Health Benefits Package Practitioners 

 Approaches to estimating CEAs, particularly their data, time and capacity requirements were not fully described in the literature. A survey of first authors of the HBP papers from the scoping review was also conducted to complement the literature.

 The survey was split into the same three sections as the data extraction. The methods section sought further detail on the methods used, eg, the number of interventions assessed; whether the scope of analysis was a full or partial HBP; and why the method was selected. It also filled gaps on which of the Welte transferability criteria were used, whether any additional transferability criteria were used, and whether any adjustments were made if analysts recalculated CERs to reduce bias.

 Surveys further requested detail on how many people conducted the assessment and how much analytical time was needed. The surveys also listed those in the international Decision Support Initiative capacity assessment questionnaire for respondents to indicate all capacities of the assessment team. Questions about data provided a full list of potential data sources collated from the literature review to validate the breadth of possible data that could be used for each method.

 The survey was piloted with one of the co-authors who was also a co-author on one of the included papers and was subsequently revised for clarity. First authors of the papers identified in the scoping review were then requested to fill in the survey in Google Forms. Where clarity was needed, brief emails were sent in follow-up. Simple descriptive statistics were used to analyze the quantitative survey data, while qualitative data was narratively summarized.

###  Step 3: Aligning the Scoping Review and Survey to aHTA

 Finally, we mapped our findings against the aHTA frame, adapting for new categories of methods where necessary. Our findings are summarized in a table of aHTA methods for CE in HBP design. Columns are split by method, and rows describe the methods’ characteristics, time and capacity requirements, and data used.

 To define the methods columns, we compared the definitions of the five aHTA methods from the systematic review to the names and summaries of methods extracted from the HBP papers. Where possible, we matched aHTA methods to HBP methods. If the HBP method did not match the five categories, we added a new category based on the most common name used in the literature. If an aHTA method was not reported in the HBP literature, we excluded it.

 The first block of the table includes a summary of each method, drawn from the literature and the survey. This includes a brief explanation of the method; potential types of assessment; and whether transferability criteria or CER adjustments are used.

 The next rows report time and capacity requirements, and potential data sources for each method. Time estimates are a summary from survey responses, which assume an average assessment team of three full-time equivalent staff. Capacity requirements are listed using the pre-specified skills categories. Data sources from the HBP literature were validated with the survey and listed in the table.

## Results

 We included 20 papers from the peer-reviewed literature (Table 1), from which we identified four methods for CEA in HBPs: expert opinion, review, model adaptation, and new model. Some of the 20 papers reported using more than one method concurrently. Additionally, we received 13 completed surveys from authors of the peer-reviewed literature, covering 16/20 papers; some respondents co-authored multiple papers from the same country.

**Table 1:** Papers Included

**Paper title**	**Publication Year**	**Country**	**Type**	**No. of interventions**	**Interventions Per Method**	**Team Size**	**Time**
The use of evidence-informed deliberative processes for health insurance benefit package revision in Iran	2022	Iran	Partial HBP	9	Review: 7 New model: 2	13	3-6 months
Revision of Malawi's HBP - a critical analysis of policy formulation and implementation	2024	Malawi	Full HBP	305	Review: 141 Expert opinion: 164	10	1-3 months
Using allocative efficiency analysis to inform HBP design for progressing towards UHC - proof of concept in countries seeking decision support	2021	Armenia	Full HBP	135	Review: 135	4	1-3 months
Using allocative efficiency analysis to inform HBP design for progressing towards UHC - proof of concept in countries seeking decision support	2021	Armenia, Cote d'Ivoire, Zimbabwe, Liberia, Afghanistan	Full HBP	100-245	Review: 74 Expert opinion: 171	2	<1 month
Report on developing the Liberia Universal Health Coverage Essential Package of Health Services	2022						
Lessons from the development process of the Afghanistan integrated package of essential health services	2023						
Assessing global evidence on cost-effectiveness to inform Pakistan's health benefits package	2024	Pakistan	Full HBP	170	Review: 170	5	3-6 months
Contextualization of cost-effectiveness evidence from literature for 382 health interventions for the Ethiopian essential health services package revision	2021	Ethiopia	Full HBP	1018	Review: 382 Model adaptation: 159 Expert opinion: 477	3	3-6 months
Generalised cost-effectiveness analysis of 159 health interventions for the Ethiopian essential health service package	2021						
Revision of the Ethiopian essential health service package: an explication of the process and methods used (Survey responses = 2)	2020						
The use of evidence-informed deliberative processes for health benefits package design in Kazakhstan	2022	Kazakhstan	Partial HBP	25	Review: all	5	1-3 months
Supporting the development of a health benefits package in Malawi	2018	Malawi	Full HBP	67	Review: all	2	<1 month
Evidence-informed update of Argentina's health benefit package: application of a rapid review methodology	2022	Argentina	Full HBP	164	Review: all	5	9 months-1 year
Cost-effectiveness of interventions to improve maternal, newborn and child health outcomes: A WHO-CHOICE analysis for Eastern Sub-Saharan Africa and South-East Asia	2021	Eastern SSA + SE Asia	Partial HBP	37	Model adaptation: all	2	>1 year
Priority setting for health service coverage decisions supported by public spending: experience from the Philippines	2018	Philippines	Full HBP	48	Model adaptation: all	20	<1 month
Reflections on the use of the World Health Organization (WHO) OneHealth tool: implications for health planning in low- and middle-income countries (LMICs)	2018						
Supporting the revision of the health benefits package in Uganda - a constrained optimization model	2023	Uganda	Full HBP	120	Review: all	3	1-3 months

Abbreviations: HBP, health benefits package; UHC, universal health coverage; SSA, Sub-Saharan Africa; SE, South-East; WHO-CHOICE, WHO CHOosing Interventions that are Cost-Effective.

 The narrative synthesis of our detailed findings are summarized in Table 2 and elaborated below. It includes an overview of the four resulting methods; time and capacity required for each; data requirements; and example countries. The full data extraction from the literature and anonymized survey responses are included in Supplementary file 1.

**Table 2:** Methods for Cost-Effectiveness in Health Benefits Package Design

	**Expert Opinion**	**Review**	**Model Adaptation**	**New Model**
Methods	Elicit expert opinion on CERs	Review and select published/estimated/synthesized CERs using pre-specified transferability criteria^a^	Estimate CERs inputting context-specific data to existing models (eg, WHO-CHOICE) and employing specific methods for reducing bias^a^	Conduct de novo CE modelling for each intervention
Scope	Assessment of full HBP, partial HBP, or few interventions^b^			Assessment of few interventions
Time^c^	1-3 months	1-10 months	1-8 months	7-13 months
Capacity	HBP design; clinical/medical expertise; public health expertise	HBP design and evidence synthesis	HBP design and health economics/econometrics, including CE modelling and budget impact analysis	
Data	Expert opinion	Peer-reviewed literature (MEDLINE, EMBASE, PubMed); meta-analyses; Tufts CEA registry; DCP; WHO-CHOICE regional estimates; other countries’ HBPs	Local, regional, global, and default unit costs, resource use, coverage, burden of disease, effectiveness	Local unit costs; local resource use; local coverage rates; local, regional, and global effectiveness evidence
Countries using these methods	Armenia, Cote d’Ivoire, Ethiopia, Liberia, Zimbabwe	Argentina, Armenia, Cote d’Ivoire, Ethiopia, Ghana, Iran, Kazakhstan, Liberia, Malawi, Pakistan, Philippines, Uganda, Zanzibar, Zimbabwe	Ethiopia, Philippines, Sub-Saharan Africa/Southeast Asia	Iran, Thailand

Abbreviations: CER, cost-effectiveness ratio; HBP, health benefits package; DCP, disease control priorities; WHO-CHOICE, World Health Organization CHOosing Interventions that are Cost-Effective; CEA, cost-effectiveness analysis. ^a^Specific transferability criteria and methods for reducing bias are elaborated in the text. ^b^Assessment of full and partial HBP could include a broad set of interventions across conditions, sometimes up to 500. Assessment of few interventions typically includes a one or a few interventions within a condition. For detail on the typology, see Baltussen et al.^3^ ^c^The time for each method assumes an average team of three full-time equivalents for comparability; it also reflects the number of interventions assessed, where many more interventions are assessed using the methods to the left of the frame, and fewer to the right.

###  Methods and Scope 

 We identified three methods that roughly aligned to aHTA categories: review (n = 12/20), model adaptation (called “transfers” in aHTA terms) (n = 6/20), and new model (called “rapid CEA” in aHTA terms) (n = 2/20). One additional method was unique to HBPs: expert opinion (n = 3/20). We excluded two aHTA methods: “de-facto” HTA and manufacturer-led submissions, as we did not find any evidence of these in the HBP literature.

 “Expert opinion” elicits experts’ opinion on the best estimate of CERs. This can be done in multiple ways. A simple approach is to set all missing CERs equal to the threshold. This was done by analysts using DCP, because all interventions in DCP are considered cost-effective, and thus this was deemed a reasonable assumption. Alternatively, a standard process of structured expert elicitation can be undertaken through a survey.

 The “review” method reviews and selects published, estimated, or synthesized CERs. Sources for each are further described under *Data* below. It then uses pre-specified transferability criteria to select the best CERs for the country under study. Survey responses confirmed common use of the Welte knock-out criteria (geographic relevance, relevance of the intervention and comparator, quality), as well as preference to newer studies and similarity of health systems in some cases.

 “Model adaptation” estimates CERs by inputting context-specific data to pre-existing CERs or models. To adjust CERs, analysts have re-calculated CERs using local costs; reduced all CERs by 30% to adjust for implementation conditions; or adjusted CERs through validating pre-populated disease burden, spending, and impact. To model new CERs, context-specific data is input to existing models (eg, WHO-CHOICE).

 “New model” builds a *de novo* CE model or analysis, with the specific method depending on the type of intervention.

 Importantly, the aHTA methods are not mutually exclusive. For example, gaps from review have been filled with expert opinion; review has been conducted alongside new model for a small set of select interventions; or review and model adaptation have been conducted together with gaps filled by expert opinion. Additionally, different combinations of expert opinion, review, and model adaptation have been used for a full HBP or partial HBP, whereas new models have been built for very few interventions.

###  Time

 The four methods vary substantially in analytical time. Many interventions have been assessed using expert opinion in one to three months, whereas one or two interventions have been assessed using new model in seven to thirteen months.

###  Capacity

 Survey respondents indicated that irrespective of method, the most common university-level skills in assessment teams included health economics or econometrics, medicine, and public health. The most common applied skills included CE modelling, budget impact analysis, and HBPs. Our interpretation of capacities which are specific to each method are reflected in Table 2.

###  Data 

 Data availability was the most important aspect of selecting methods according to survey respondents, in comparison to relevance and quality of data obtained; time; and capacity. It is a required input for the review, model adaptation, and new model methods. Sources of data for each are summarized below.

####  Review

 Several databases, types of synthesized evidence, and meta-analyses are available for review. Databases offer the most comprehensive resource for CERs, whereas synthesized evidence and meta-analyses are focused on a smaller subset of interventions.

 Databases used for HBPs include the Tufts CEA registry and peer-reviewed literature databases. The Tufts CEA registry offers a database of more than 12 000 CEAs. It includes studies that exclusively use quality-adjusted life years or disability-adjusted life years as measures of effectiveness, rather than disease-specific or study-specific measures, to ensure comparability across interventions.^7^ Pre-extracted CERs and other key aspects from the CEA studies are available for download from Tufts. MEDLINE, EMBASE, and PubMed are the peer-reviewed databases most used in HBP design, and also offer non-disability-adjusted life years or quality-adjusted life year studies.

 The most common resource for synthesized CE estimates for HBPs is DCP. DCP provides values for 218 interventions considered “essential” for LMICs. Estimates reflect a systematic review of global CE evidence, validated by hundreds of experts.^20^ WHO-CHOICE also offers synthesized evidence in the form of regional estimates. Values are produced for 20 disease areas and 500 interventions, using their suite of user-friendly models.^21-23^ While these estimates are reported in the literature as regional analyses, they are also incorporated into countries’ HBP assessments. Finally, there may be systematic reviews of CE for specific disease areas that could facilitate the review of CE.

 A new promising source of CE values are meta-analyses. They gather existing CE evidence on well-studied interventions and use statistical models to predict country-specific CERs. Meta analyses are currently available for HPV vaccination and rotavirus, with estimates for a set of HIV, tuberculosis, malaria, and syphilis interventions expected soon.^10-12^

####  Model Adaptation/New Model 

 For model adaptation, data sources include local, regional, or global estimates of costs, resource-use, burden of disease, and/or effectiveness. Local costs can be used to re-calculate CERs. User-friendly models for this method were limited to the WHO-CHOICE modules. To model health benefits and disease burden, it offers the following modules: the tuberculosis impact model and estimates (TIME) for tuberculosis; the lives saved tool (LiST) for reproductive, maternal, neonatal, and child health, nutrition, and water and sanitation; “FamPlan” for family planning; “OpenMalaria” for malaria; AIDS impact module (AIM) for HIV/AIDS; and the non-communicable disease impact module. Additionally, “DemProj” models population growth and other demographics; the “OneHealth” tool is pre-populated with default costing estimates; and the WHO generalized CEA tool can be used to estimate new CERs.

 For new models, local epidemiological models can be identified to pair with *de novo* CE models.

## Discussion

 Based on our findings, we have outlined several considerations that national assessment teams may wish to consider when designing HBP assessments which are elaborated below (Figure).

**Figure:**
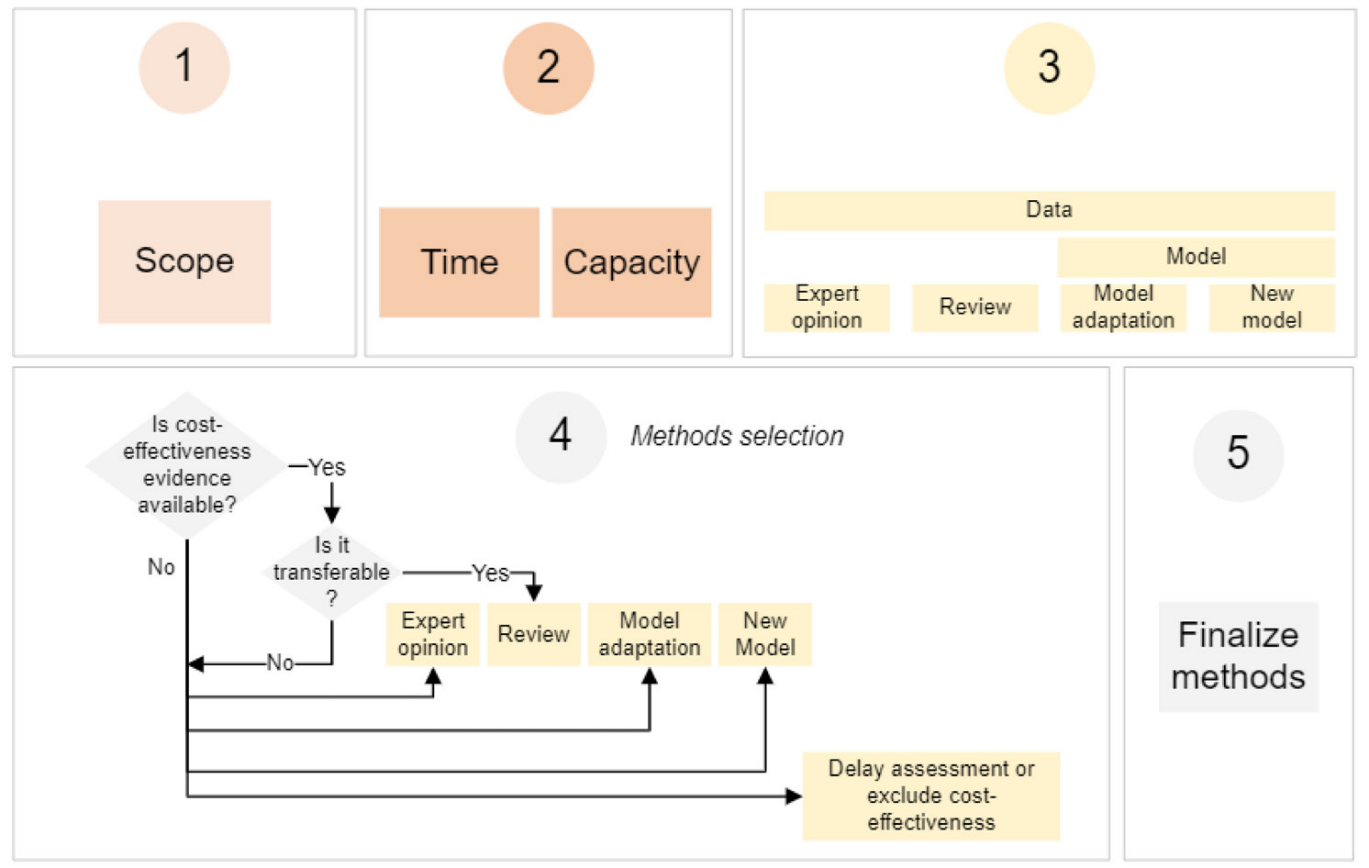
Considerations for Assessment Design.

###  1: Define the Scope 

 Whether the scope of an assessment is a full HBP or partial HBP should be agreed by the assessment team.^3^ The number of interventions and descriptions of each should be listed. Then, it is important that analysts consider whether the interventions are aligned with the nomenclature and scope of available data sources for later mapping. For example, if the assessment will use DCP or WHO-CHOICE, analysts can review whether alignment of local interventions with these resources is possible. If not, other structures can be considered for alignment such as standard coding systems like the International Classification of Disease and the accompanying International Classification of Health Interventions or the WHO UHC-Compendium.^24,25^

 Interventions for HBPs are defined in many ways which is inherently a difficult part of HBP design, and thus mapping to existing lists and data is an important first consideration. The only exception is where the scope is instead an incremental analysis of a single intervention, which would not requirement alignment with existing data but rather would be defined using a standard scoping framework for economic evaluation such as population, intervention, comparator, outcome, or “PICO.”^26^

###  2: Time and Capacity 

 The amount of time available for CE assessment should also be considered with either the government or social insurer, as part of broader time expectations for the assessment of all criteria and subsequent appraisal.

 The capacity of the team will also be linked to the type of method which is feasible. While survey responses were nearly the same about technical and applied skills needed across methods, there are important variations. All methods arguably require an understanding of HBP design. For expert opinion, additional skills may be focused on clinical, medical, or public health expertise. For review, analysis mostly requires evidence synthesis skills, such as systematic reviewing. And for model adaptation or new models, further health economics expertise is required including CE modelling and budget impact analysis.

 The available capacity of any additional contributors to the assessment can also be considered and documented. For example, in expert opinion, there is a need for local experts willing to participate in elicitation. In model adaptation and new model, a local modeler may need to be recruited to the assessment team depending on the disease area in scope.

###  3: Define Data and Models by Method

 Available data and models for assessment vary considerably depending on the types of interventions being assessed. It may require some initial work to identify all stakeholders who are actors in the service area(s) to ensure a comprehensive review of data availability.

 The review of data availability may be sequenced in three stages. First, an initial review of possible global data sources for review and model adaptation can be undertaken. Potential sources are listed in Table 2, though additional sources may be available depending on the scope of analysis. Importantly, analysts may wish to consider whether global sources contain ICERs or average cost-effectiveness ratios (ACERs). In our reporting, we have intentionally referred to ‘CERs’ generally, as using ICERs or ACERs is a hotly debated topic which is elaborated in detail elsewhere.^27^ While ACERs are considered preferred for ensuring allocative efficiency of HBP design, the global literature contains a mix of ACERs and ICERs, the implications of which should be considered by the assessment team. Second is a review of available national data. This could include identifying available CE studies or data on CE parameters (eg, unit costs, resource use, burden of disease, health effects) for the topic under consideration. These could be used for the model adaptation method to either recalculate CERs or to input to WHO-CHOICE modules.

 Finally, it is worth exploring whether any available global or local models are available. This could include whether WHO-CHOICE models are available for the disease being studied, and whether any national epidemiological models or CE models on the topics under study are available. If new model is selected as an assessment method, further identification of national and global data will be required to parameterize the model. For example, availability of data for the CE parameters can be reviewed, as well as methods for filling data gaps.

###  4: Methods Selection 

 In reviewing the scope, time, data, and capacity, several considerations can be made for methods selection. In the first instance, the interaction between time and scope is important to consider. While a full HBP could be assessed using any combination of expert opinion, review, and model adaptation, it would be impossible to do the same using new model for the same number of interventions. If an assessment seeks to consider many interventions at once, this may automatically eliminate new model as a methodological choice.

 Next, since the majority of HBP papers we identified used the review method (n = 12/20), methods selection may begin by considering whether CE evidence is available and transferable to the national context. This requires conducting a rapid review of availability literature to document roughly what literature exists in the area, and whether the interventions appear to be relevant to local context. This is not a full “review.” Rather, it is a quick check of the listed resources to understand approximately how many CERs are available on the topic, whether there are any studies from similar geographic contexts, and whether the interventions in those studies are similar to those being delivered in the study setting.

 If the global data are broadly available and transferable, then the main method of choice may be review. The availability of CE evidence on the topics of analysis in each of the data sources is likely to narrow the list of data sources analysts use. If CE evidence is only available in one of the data sources (Tufts, DCP, WHO-CHOICE, meta-analysis, and systematic reviews), analysts can default to using this source. If CE evidence is available in multiple data sources, additional considerations should be made. For example, it may still be preferred to maintain the simplicity of using only one data source with the most CE evidence, which is likely to be Tufts. Or analysts may prefer to focus on interventions and evidence which are considered by many experts to be CE in LMICs, in which case DCP is a better choice. Another consideration is whether any adjustments to CERs will be made, for which the Tufts downloadable data is helpful. For example, if local costs are available, analysts may wish to recalculate CERs using effects extracted from studies in Tufts. Alternatively, if regional estimates of CERs are considered sufficient, WHO-CHOICE may be preferred. Finally, analysts could also create a hierarchy of evidence; for example, the small pool of meta-analyses may be reviewed first to prioritize predicted country-specific CERs, and then a review conducted of one of the other broader data sources.

 If the data identified under review are not available or deemed not transferable, options include using one of the other three methods; delaying the assessment; or excluding a CE assessment for some interventions. In doing so, there are trade-offs. Expert opinion takes very little time and only requires deciding an approach for eliciting expert opinion and convening experts. It is a tested approach to “fill gaps” when CERs are missing in HBP assessments that analyze many interventions at once. Alternatively, a model adaptation is possible using the WHO-CHOICE modules, but this requires an understanding of or willingness to learn how to use these models. More information on the utility of these tools and others to support HBP design is reported in a recent framework on resource allocation tools.^28^ A new model requires far more time and substantial modelling expertise. If this is available locally, it may be a favorable option for building capacity and calculating nationally-relevant estimates. However, it should be weighed against the available time and envisioned scope of assessment. If an assessment seeks to evaluate 100 interventions in less than a year, this option may not be feasible. A final option is to delay assessment or not assess CE for some interventions. The latter is an alternative to filling gaps using expert opinion, and has been done in some HBP assessments that used review but did not find CERs for all interventions assessed.

 Additional consideration should be given to whether local decision-makers will be willing to make a recommendation based on the evidence presented. They may be comfortable making recommendations based on expert opinion for the topic(s) in scope, or may prefer to wait until more detailed, nationally-relevant data and analysis are available.

 After exploring these considerations, a national assessment team should be equipped to compose draft assessment methods. If more than one option is available, it may be useful to summarize each option in terms of its time, data, and capacity needs.

###  5: Finalize Methods

 Finally, agreeing the methods through consensus by a group of key stakeholders may be valuable in setting expectations for the future HBP assessment. This could include the local HBP committee, government stakeholders, clinical experts, and the assessment team. Any adjustments to the scope, time, and capacity required can be discussed and deliberated. Interventions may be assigned to different methods depending on data availability, and others may be postponed for future assessments. Depending on the perceived quality of the evidence to be used, the group could also consider how this will be reflected when the evidence is presented for appraisal.

 Importantly, these are all only considerations which may not fully reflect the shifting dynamics in country. Completing an HBP assessment can be facilitated by strong leadership from a government institution and/or leading agency. It also requires careful balancing of government ownership and development partner involvement as well as building capacity of local teams to prioritize HBPs independently in the future. Thus, these considerations are likely to shift in any given context.

###  Summary 

 Our work is the first attempt to classify aHTA methods for CEA in HBPs. It articulates four methods used in HBPs for obtaining CEA values—expert opinion, review, model adaptation, and new model—and the scope; time and capacity; and data for each.

 The primary audience of this work is HBP practitioners, including academics and policy-makers, in any country in need of practical guidance for selecting assessment methods. It is not meant to be prescriptive. Rather, it should be tested as an aid to review existing methods for CE assessment in HBPs in a structured way. In doing so, it could help ensure national ownership and enable transparent methodological choices to be presented to appraisal committees. Using such an approach is also an opportunity for practitioners to reflect and provide feedback to producers of CEAs on how to design their work in a way that supports HBP design.

 Through experience, the table and guidance presented here should be adapted and expanded. For example, our present work only considers transferability of existing data or adjustments made to CERs. It does not dig into deeper issues of bias, uncertainty, transparency, or risk of the different estimates from key sources. Further development of the framework could consider these issues to establish an approach for estimating the risk of making the wrong decision by using different aHTA methods, which can better inform methods selection. Additionally, the guidance contained here focuses on CE as a central criterion for HBP design, but CE is not the only important criterion. Future work should consider potential methods for assessing additional prioritization criteria such as budget impact, equity, and feasibility, and the time, data, and capacity requirements for assessing multiple criteria simultaneously. Finally, our findings did not identify pros and cons of each of the methods, which could be further elaborated in future research.

 It is possible that we missed some CEA methods due to focusing on what *has been* done for HBPs and is reported in the literature, but not what *could be* done. The rapid nature of our scoping review restricted us to papers labelled as HBP assessments, but we may have found additional methods, if we had broadened our scope to other priority setting exercises. However, as we wanted to ensure we reflected on methods choice in the context of existing HBP resources, we consider our scoping approach to be fit-for-purpose. Moreover, because our reporting is based on what has previously been done in HBP, using the aHTA framing has not yet been fully tested in a live policy context. Further refinement of our findings and considerations would benefit from countries that have tested our approach.^27^

## Conclusion

 Reviews of HBPs have been ongoing since the 1993 World Bank report.^29^ While 30 years have passed, the development of resources to support CEA for HBPs has been disproportionately driven by technical partners. It is time to shift away from focusing on these resources to a nationally-owned process for selecting context-appropriate methods, and it is our hope that this paper is a first step to supporting this approach.

## Acknowledgments

 The authors would like to thank Francis Ruiz and Andres-Madriz Montero from the London School of Hygiene and Tropical Medicine for their early review and testing of the framework. Additionally, we would like to thank the survey respondents for their participation; their responses added valuable context to our paper.

## Ethical issues

 This study was approved by the London School of Hygiene and Tropical Medicine’s ethics committee, reference number 28265.

## Conflicts of interest

 Authors declare that they have no conflicts of interest.

## Supplementary files


Supplementary file 1 contains Table S1 (the search strategy).
